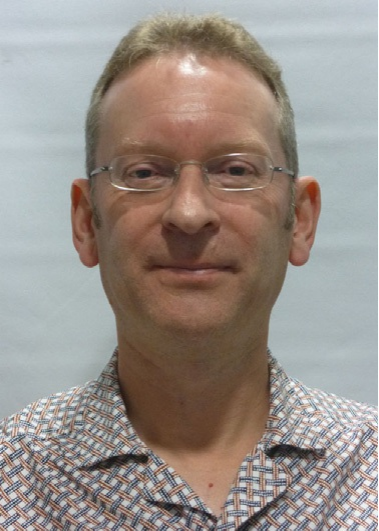# Control of Absolute Stereochemistry in Transition‐Metal‐Catalysed Hydrogen‐Borrowing Reactions

**DOI:** 10.1002/chem.202001253

**Published:** 2020-09-11

**Authors:** Timothy Kwok, Oskar Hoff, Roly J. Armstrong, Timothy J. Donohoe

**Affiliations:** ^1^ Chemistry Research Laboratory University of Oxford Oxford OX1 3TA UK

**Keywords:** amines, asymmetric, enantioselective, enolates, hydrogen borrowing

## Abstract

Hydrogen‐borrowing catalysis represents a powerful method for the alkylation of amine or enolate nucleophiles with non‐activated alcohols. This approach relies upon a catalyst that can mediate a strategic series of redox events, enabling the formation of C−C and C−N bonds and producing water as the sole by‐product. In the majority of cases these reactions have been employed to target achiral or racemic products. In contrast, the focus of this Minireview is upon hydrogen‐borrowing‐catalysed reactions in which the absolute stereochemical outcome of the process can be controlled. Asymmetric hydrogen‐borrowing catalysis is rapidly emerging as a powerful approach for the synthesis of enantioenriched amine and carbonyl containing products and examples involving both C−N and C−C bond formation are presented. A variety of different approaches are discussed including use of chiral auxiliaries, asymmetric catalysis and enantiospecific processes.

## Graphical abstract

**Borrowing hydrogen**: This Minireview summarizes the rapidly developing area of transition‐metal‐catalysed asymmetric hydrogen‐borrowing catalysis. A range of enantioselective processes involving alkylation of both amines and enolates are presented. A wide variety of strategies have been employed to induce asymmetry in these reactions including asymmetric reduction, dynamic kinetic asymmetric transformation and incorporation of chiral auxiliaries.

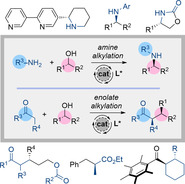

## Introduction

**Scheme 1:**
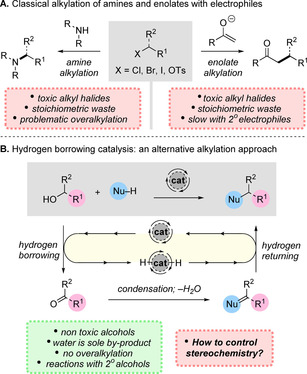
Contrasting classical alkylation of amines and enolates with hydrogen‐borrowing catalysis.

Alkylation is a fundamental process in organic synthesis and is typically amongst the first reactions taught to chemistry undergraduate students.^1^ A wide variety of amine and enolate nucleophiles can be employed, enabling C−N or C−C bond formation with halide or pseudohalide electrophiles (Scheme 1 A). However, in practice, this process suffers from significant drawbacks. For example, the electrophiles employed are typically highly toxic and the reactions produce stoichiometric quantities of waste which must be separated after the reaction.^2^ Moreover, the alkylation of amines is often complicated by the formation of over‐alkylated side‐products.^3^ In the case of enolate alkylation, reactions with primary electrophiles typically proceed efficiently, but the analogous reactions of secondary electrophiles are often sluggish and can be hampered by competing elimination processes. Hydrogen‐borrowing catalysis has emerged as a powerful alternative alkylation strategy, which solves many of these problems (Scheme 1 B).^4^ This approach relies on a catalyst that oxidizes the alcohol starting material to the corresponding carbonyl compound, temporarily storing the hydrogen which is produced. The aldehyde or ketone then condenses with the nucleophilic partner (usually an amine or enolate), eliminating water to form an intermediate which is finally reduced by the stored hydrogen to form the alkylated product and regenerate the active catalyst. This reaction produces water as the sole by‐product and enables non‐activated alcohols to serve as electrophiles, thereby negating the requirement for toxic alkyl halide electrophiles. Using this approach, amines can be reacted without over‐alkylation and secondary alcohols can be employed. However, a key question still remains, namely, how can absolute stereochemistry be controlled in such a reaction? Recently, a number of reports have emerged that address this important challenge and this is the focus of this Minireview.^5^

**Scheme 2:**
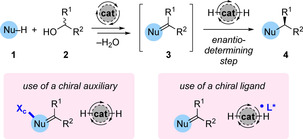
General strategies to achieve asymmetric hydrogen‐borrowing alkylation with secondary alcohols.

The majority of asymmetric hydrogen‐borrowing processes that have been developed involve reactions between a nucleophile **1** and a racemic secondary alcohol **2** bearing two different groups (R^1^ ≠ R^2^). This process results in the formation of a chiral product **4** (Scheme 2), the stereochemistry of which is established during the step in which hydrogen is returned to achiral intermediate **3**. This provides a unique opportunity to induce asymmetry by controlling the facial selectivity of the reduction step. In practice, two distinct strategies can be envisaged to direct such a process: (i) diastereoselective reduction of an enantiopure substrate, for example, by introduction of a chiral auxiliary; (ii) addition of an external chiral ligand (L*) which mediates a catalytic asymmetric reduction. Highly enantioselective hydrogen‐borrowing processes which employ both of these strategies have been developed and will be discussed in this Minireview. Additionally, several related processes will be presented which enable stereochemistry to be controlled at sites adjacent to the reacting alcohol, for example via dynamic kinetic asymmetric transformation or enantiospecific alkylation.

Overall, the aim of this Minireview is to present the current state of the art in transition‐metal mediated asymmetric hydrogen‐borrowing catalysis. The focus is upon processes that form a new C−C or C−N bond and examples involving asymmetric transfer hydrogenation or redox shuttles are not covered. A wide variety of approaches to achieve stereocontrol will be discussed including the use of chiral auxiliaries, transition‐metal catalysis and enantiospecific processes.

## C−N Bond Formation

**Scheme 3:**
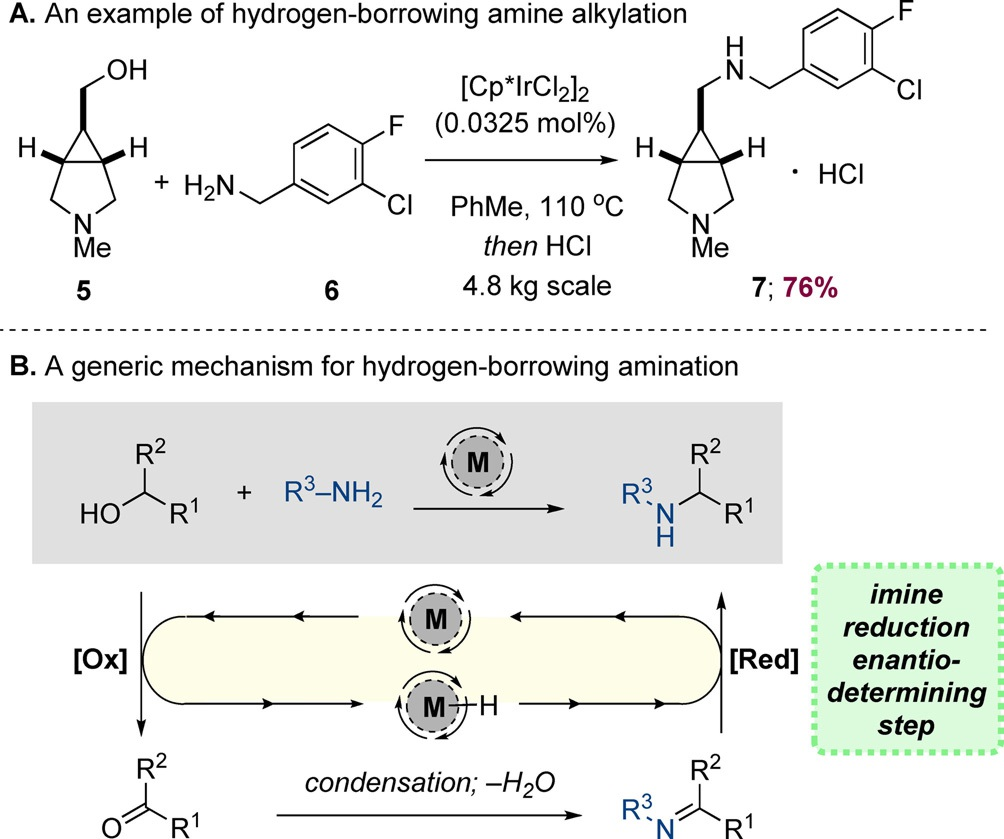
A representative example of amine alkylation using hydrogen‐borrowing catalysis and a generic mechanism for C−N bond formation.

Amines are hugely important building blocks, with extensive applications in materials science, natural product synthesis and in the pharmaceutical industry. Hydrogen‐borrowing catalysis is a well‐established method for N‐alkylation and is widely employed for the synthesis of important racemic and achiral amine containing materials. As an illustrative example of a hydrogen‐borrowing process which targets an achiral product, Pfizer have disclosed a kilogram‐scale hydrogen‐borrowing alkylation between alcohol **5** and substituted benzylamine **6** (Scheme 3 A).^6^ This process was catalysed by 0.0325 mol % of [Cp*IrCl_2_]_2_, producing amine **7** in 76 % yield, which could be converted to the anti‐schizophrenic medicine PF‐03463275 in a single step. Mechanistically, this type of reaction proceeds by metal‐mediated oxidation of the alcohol to give the respective ketone or aldehyde as well as a metal hydride species (Scheme 3 B). The carbonyl then undergoes condensation and the resulting imine is reduced by the metal hydride to give the amine and the regenerated metal catalyst.

In order to carry out an asymmetric amination reaction, the facial selectivity of the final reduction of the imine (or iminium) intermediate must be controlled. This section will discuss three main strategies to induce asymmetry in this chemistry, namely: (i) Use of chiral substrates; (ii) Use of chiral ligands in transition metal catalysis; and (iii) Use of enzymes. These strategies are detailed sequentially in the following sub‐sections.

### Diastereoselective reactions with chiral substrates

#### Enantiopure amines

**Scheme 4:**
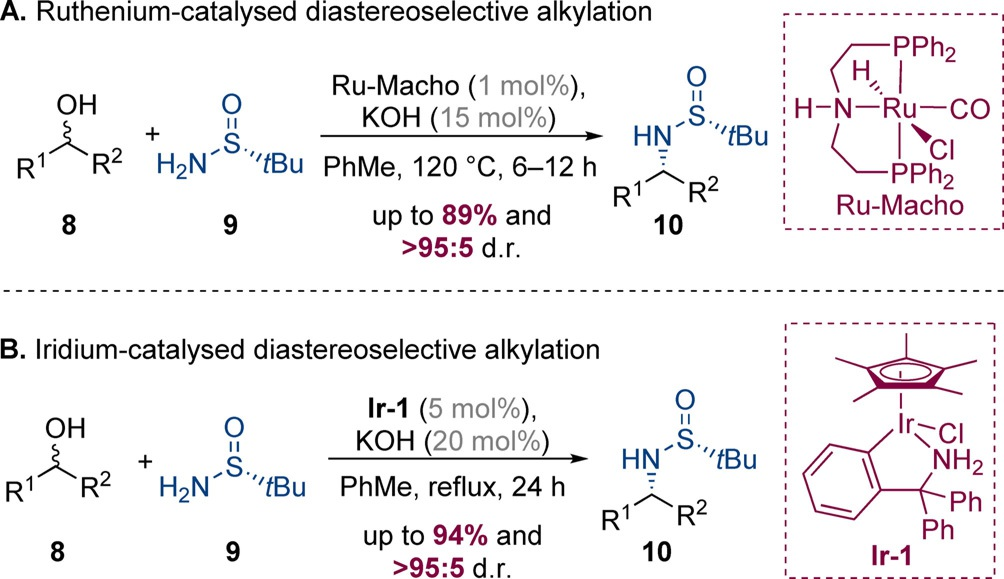
Asymmetric hydrogen‐borrowing reactions involving Ellman's *tert*‐butanesulfinamide auxiliary.

Various chiral amine nucleophiles have been used to induce diastereoselectivity in hydrogen‐borrowing alkylation processes and Ellman's chiral *tert*‐butanesulfinamide auxiliary has proved to be particularly effective in this context. This strategy was pioneered by Dong, Guan, and co‐workers in 2014, who employed a commercially available ruthenium(II) PNP‐type pincer catalyst (Ru‐Macho) to achieve diastereoselective alkylation with a range of benzylic and aliphatic secondary alcohols (Scheme 4 A).^7^ A difference in steric bulk between the two groups flanking the secondary hydroxyl group was required for good diastereoselectivity—a trend consistent with that observed by Ellman and others in the reduction of sulfinylimines.^8^ Very high diastereoselectivity was achieved for the majority of alcohol substrates, which is particularly impressive considering the high reaction temperature (120 °C). Notably, no epimerization of the chiral sulfinimide was observed, enabling the synthesis of the alkylated products in enantiopure form. Subsequently, Xia, Zhang, and co‐workers developed a related process mediated by iridium catalyst **Ir‐1** (Scheme 4 B).^9^ A range of secondary alcohols underwent highly diastereoselective alkylation, including relatively hindered examples such as 1‐phenyl‐1‐propanol. It was also demonstrated that the chiral auxiliary could be cleaved to obtain the corresponding enantiopure primary amines.

**Scheme 5:**
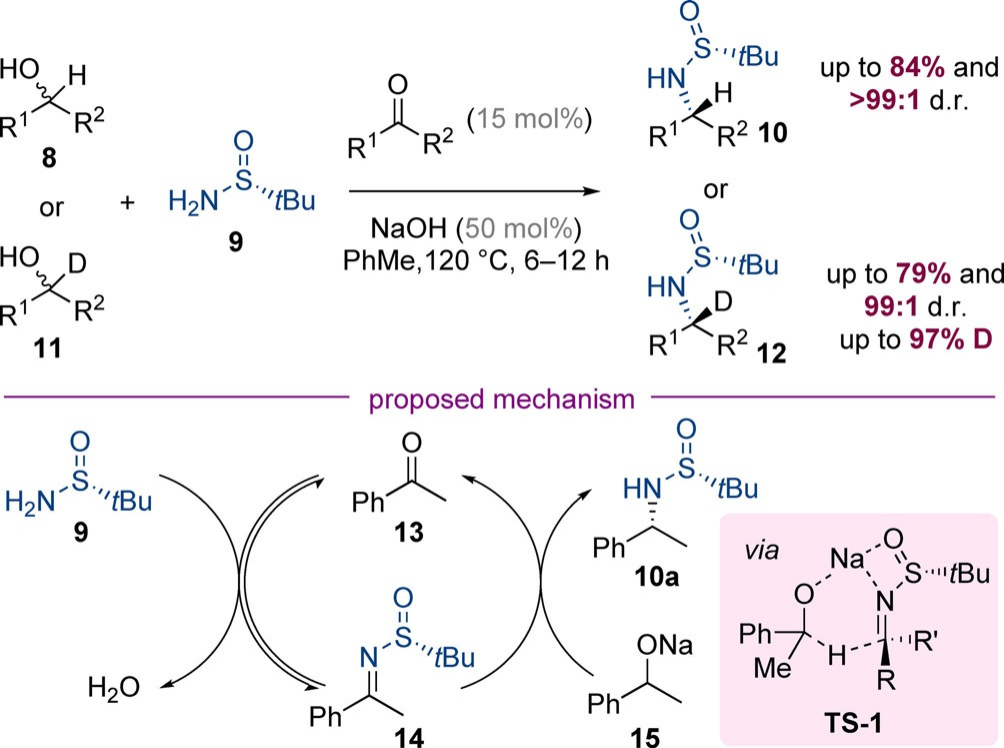
Transition‐metal‐free alkylation controlled by Ellman's auxiliary.

In the same year, Lei, Xiao, Wang, and co‐workers made the surprising discovery that a similar alkylation reaction can be conducted in the absence of any transition‐metal catalyst (Scheme 5).^10^ It was shown that by instead adding a small quantity (15 mol %) of the ketone which would result from alcohol oxidation, highly diastereoselective N‐alkylation was observed. The method was also used to prepare enantioenriched deuterium‐labelled amines **12** from the corresponding racemic α‐deuterated alcohols. The products were isolated with very high levels of deuterium incorporation. The authors suggest that the mechanism of this process is initiated by condensation of amine **9** with ketone **13**. The proposed key step involves Meerwein–Ponndorf–Verley‐type reduction of the resulting imine **14** by sodium alkoxide **15** via a chelated transition state **TS‐1**. This hypothesis is supported by the observation that the reaction progressively stalls when increasing quantities of 15‐crown‐5, a crown ether known to sequester Na^+^, are added.

**Scheme 6:**
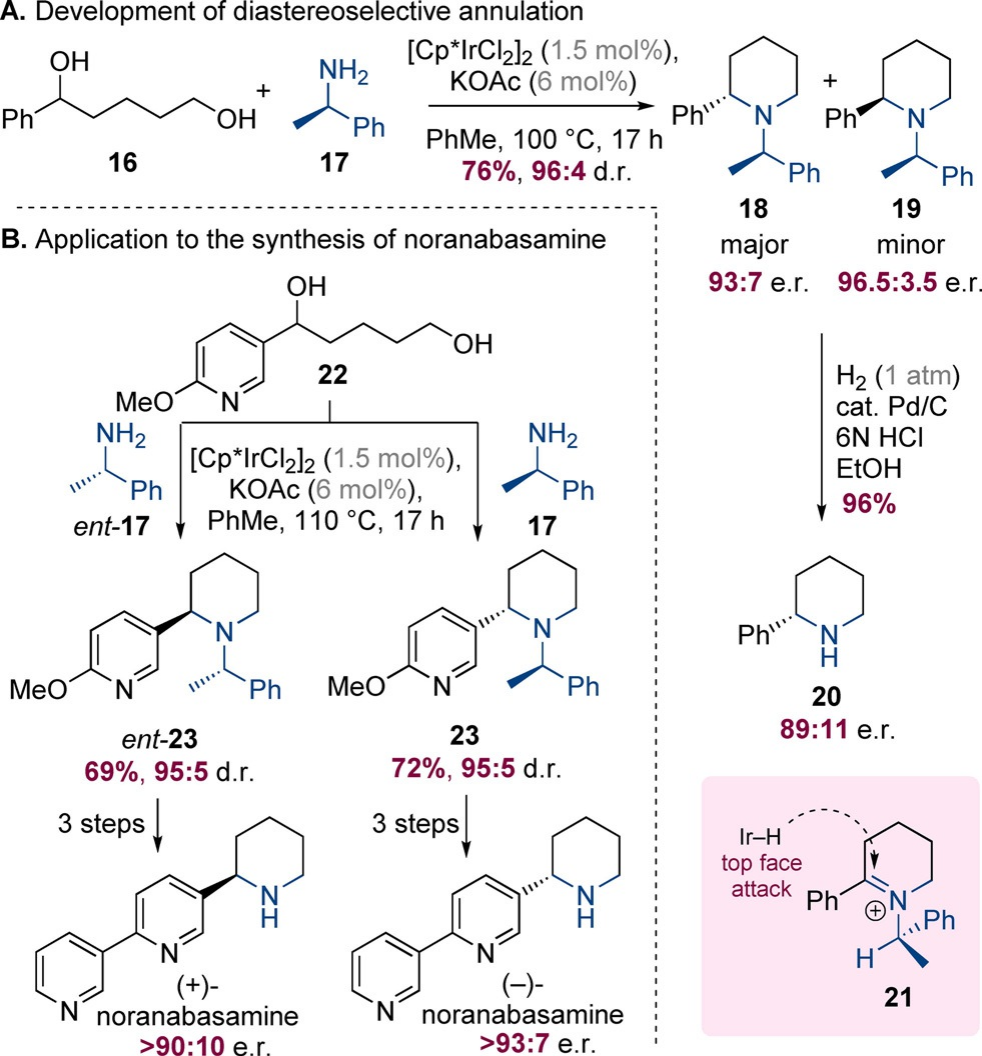
Diastereoselective hydrogen borrowing with α‐methylbenzylamine.

Yamaguchi, Fujita and co‐workers have reported that enantiopure (*R*)‐α‐methylbenzylamine (99.5:0.5 e.r.) can undergo double hydrogen‐borrowing alkylation with a diol to form piperidine **18** (Scheme 6 A).^11^ The product was formed in 96:4 d.r. with a slight erosion in enantiopurity (93:7 e.r. and 96.5:3.5 e.r. for the major and minor diastereomers, respectively). The major diastereomer was suggested to have arisen from hydride delivery to the less hindered face of intermediate **21**; the small amount of racemization was attributed to benzylic deprotonation of this intermediate. The benzyl group readily underwent hydrogenolysis to unveil the secondary amine **20**. This strategy was later employed by Trudell and co‐workers as the key step in a total syntheses of noranabasamine (Scheme 6 B).^12^ Diol **22** was prepared in two steps and then subjected to Yamaguchi's hydrogen‐borrowing annulation together with either **17** or *ent*‐**17** to separately give either **23** or *ent*‐**23**. These intermediates could be converted to (−)‐ or (+)‐noranabasamine in three steps.

**Scheme 7:**
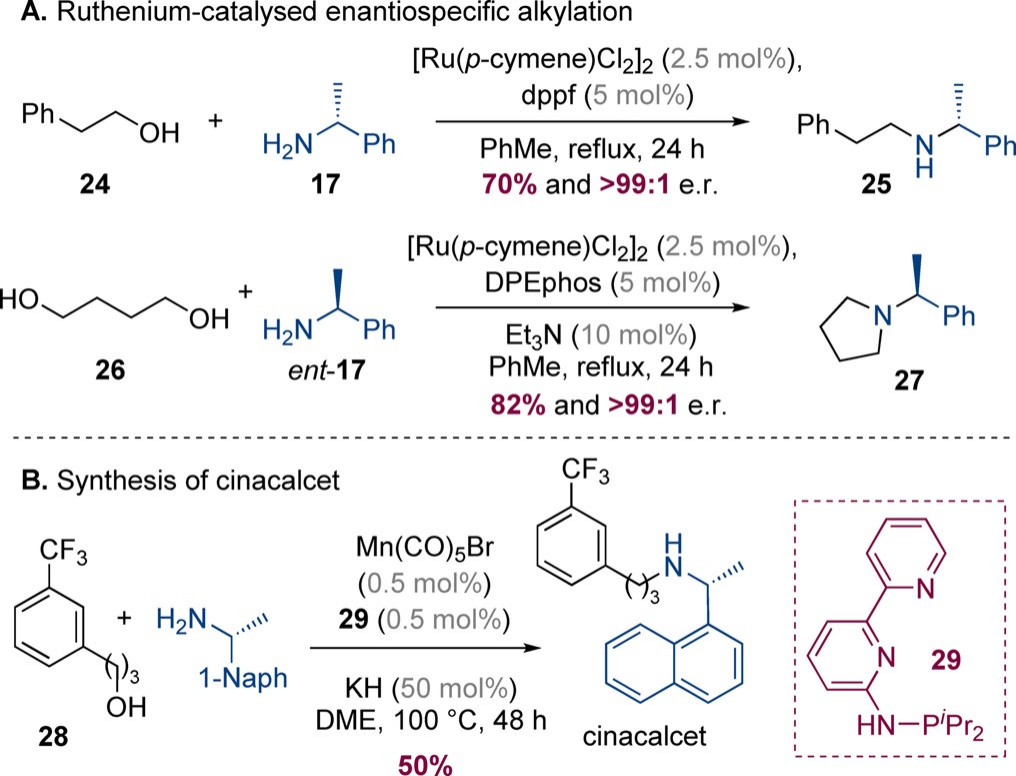
Enantiospecific hydrogen‐borrowing reactions with α‐methylbenzylamine derivatives.

Interestingly, enantiopure α‐methylbenzylamine derivatives do not always undergo racemization in hydrogen‐borrowing reactions. Indeed, Williams and co‐workers have shown that (*R*)‐α‐methylbenzylamine (**17**) can undergo hydrogen borrowing with a primary alcohol **24** under ruthenium‐catalysed conditions with complete stereochemical integrity (Scheme 7 A).^13^ A similar reaction with a 1,4‐diol also generated the corresponding piperidine **27** without racemization. A similar process has been reported by Hultzsch and co‐workers which utilized a manganese‐catalysed hydrogen‐borrowing alkylation as a key step to prepare the hyperparathyroidism medication, cinacalcet, from an enantiopure naphthyl substituted amine (Scheme 7 B).^14^

**Scheme 8:**
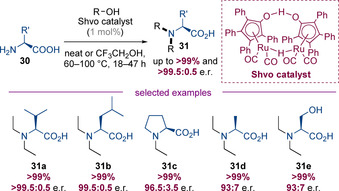
Enantiospecific hydrogen‐borrowing alkylation of unprotected amino acids with the Shvo catalyst.

Yan, Feringa, and Barta have reported a system for the N‐alkylation of unprotected amino acids with alcohols using the Shvo catalyst (Scheme 8).^15^ The choice of catalyst was essential—the Shvo catalyst is capable of bifunctional activation of the alcohol without the need for external base that could result in racemization of either the starting materials or products. A wide variety of amino acids were successfully N‐alkylated under these conditions with excellent yields. Surprisingly, even serine which bears a free hydroxyl group was a competent substrate that gave the desired product in quantitative yield. For the alkylation of alanine and serine (**31 d** and **31 e**, respectively), a small amount of racemization was observed, but in the majority of cases the products were obtained with near perfect enantiospecificity.^16^

**Scheme 9:**
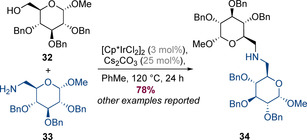
Hydrogen‐borrowing reactions with carbohydrate‐derived substrates.

Cumpstey, Martín‐Matute and co‐workers have shown that alcohols and amides which are both derived from carbohydrates can be coupled under hydrogen‐borrowing conditions to afford amino sugars such as **34** (Scheme 9).^17^ Both the amine and alcohol partners reacted without erosion of stereochemistry to afford the products with complete diastereoselectivity.

**Scheme 10:**
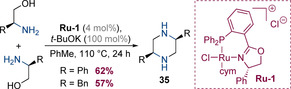
Dimerization of enantiopure 1,2‐amino alcohols.

Takacs and co‐workers have reported that in the presence of ruthenium complex **Ru‐1**, enantiopure 1,2‐amino alcohols can undergo dimerization to form *cis*‐2,5‐disubstituted piperazines **35** (Scheme 10).^18^ The products were obtained with complete diastereo‐ and enantiocontrol, suggesting that no epimerization occurred, although it is possible that the chiral catalyst may play a role in this transformation.

#### Enantiopure alcohols

**Scheme 11:**
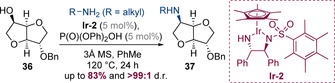
Diastereoselective hydrogen‐borrowing amination of isohexides.

Diastereoselectivity can also be induced by using chiral alcohol substrates. This strategy was employed by Jacolot, Popowycz and co‐workers to carry out iridium‐catalysed aminations of isohexides (Scheme 11).^19^ Using conditions adapted from those reported by Zhao (vide infra), highly diastereoselective alkylation could be achieved affording bicyclic amines **37**. Although a chiral catalyst was used in this process, the diastereoselectivity appears to be a result of substrate control. Indeed, during reaction optimization it was shown that complete diastereoselectivity was also observed using achiral iridium catalysts.

**Scheme 12:**
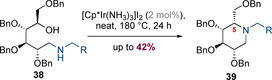
Diastereoselective cyclization of multisubstituted amino alcohols.

Chen and co‐workers have reported an intramolecular hydrogen‐borrowing alkylation of glucose‐derived 1,5‐hydroxamines (Scheme 12).^20^ The reaction proceeded in moderate yields (up to 42 %) but afforded high diastereoselectivity at the newly formed stereogenic center at C5. The relative stereochemical outcome of this process is somewhat surprising as it is not consistent with axial addition of iridium hydride to a half‐chair iminium species. The authors propose that the high temperature of the process (180 °C) may enable an alternative pathway involving a boat‐like transition state to become viable.

**Scheme 13:**
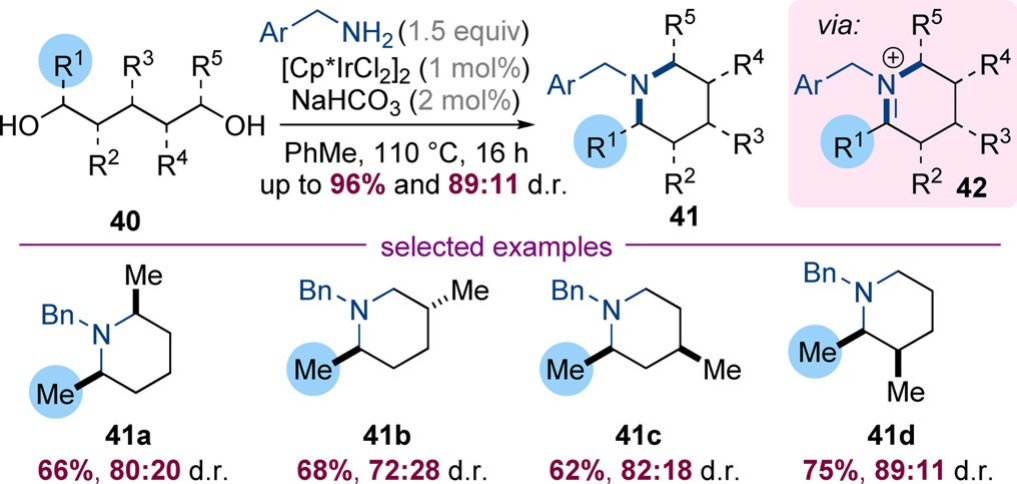
A diastereocontrolled hydrogen‐borrowing annulation reaction.

Donohoe and co‐workers have reported an iridium‐catalysed synthesis of saturated aza‐heterocycles via a hydrogen‐borrowing annulation between amines and multi‐substituted diols (Scheme 13).^21^ This reaction is thought to proceed via a mechanism involving two successive hydrogen‐borrowing alkylations, the final step of which would be the reduction of a cyclic iminium species **42**. The diastereoselectivity of this process was systematically investigated by introducing a second substituent at each possible position around the heterocyclic core leading to piperidines **41 a**–**d** in high yields. For **41 a**–**c** the relative stereochemistry is consistent with axial addition of iridium hydride to a half‐chair bearing an equatorial (or pseudoequatorial) substituent.^22^ For **41 d**, it was proposed that A^1, 3^ strain forces the methyl group into a pseudoaxial conformation leading to the *cis*‐diastereoisomer.

**Scheme 14:**
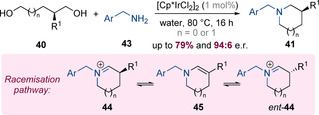
An enantiospecific hydrogen‐borrowing annulation reaction.

In the same work, the authors also developed a synthesis of enantioenriched C3‐substituted pyrrolidines and piperidines from enantiopure 1,4‐ and 1,5‐diols (Scheme 14).^21^ Under typical conditions employed for the iridium‐catalysed amine alkylation, significant racemization was observed, presumably as a consequence of deprotonation of cyclic iminium intermediate **44** to the corresponding enamine **45**. By carrying out the reaction in water, this undesired racemization pathway could be almost entirely suppressed enabling the isolation of highly enantioenriched C3‐substituted piperidines and pyrrolidines.

### Use of chiral additives and transition‐metal catalysts

Asymmetric transition metal catalysis has also been widely used as a strategy to achieve asymmetric hydrogen borrowing. The most common of examples involve control of the same stereocenter at which the new C−N bond is formed. Other examples involve control of a stereocenter that is remote to the new C−N bond being formed. These two sub‐classifications will be discussed separately.

#### Controlling the stereocenter at which the C−N bond is formed

**Scheme 15:**
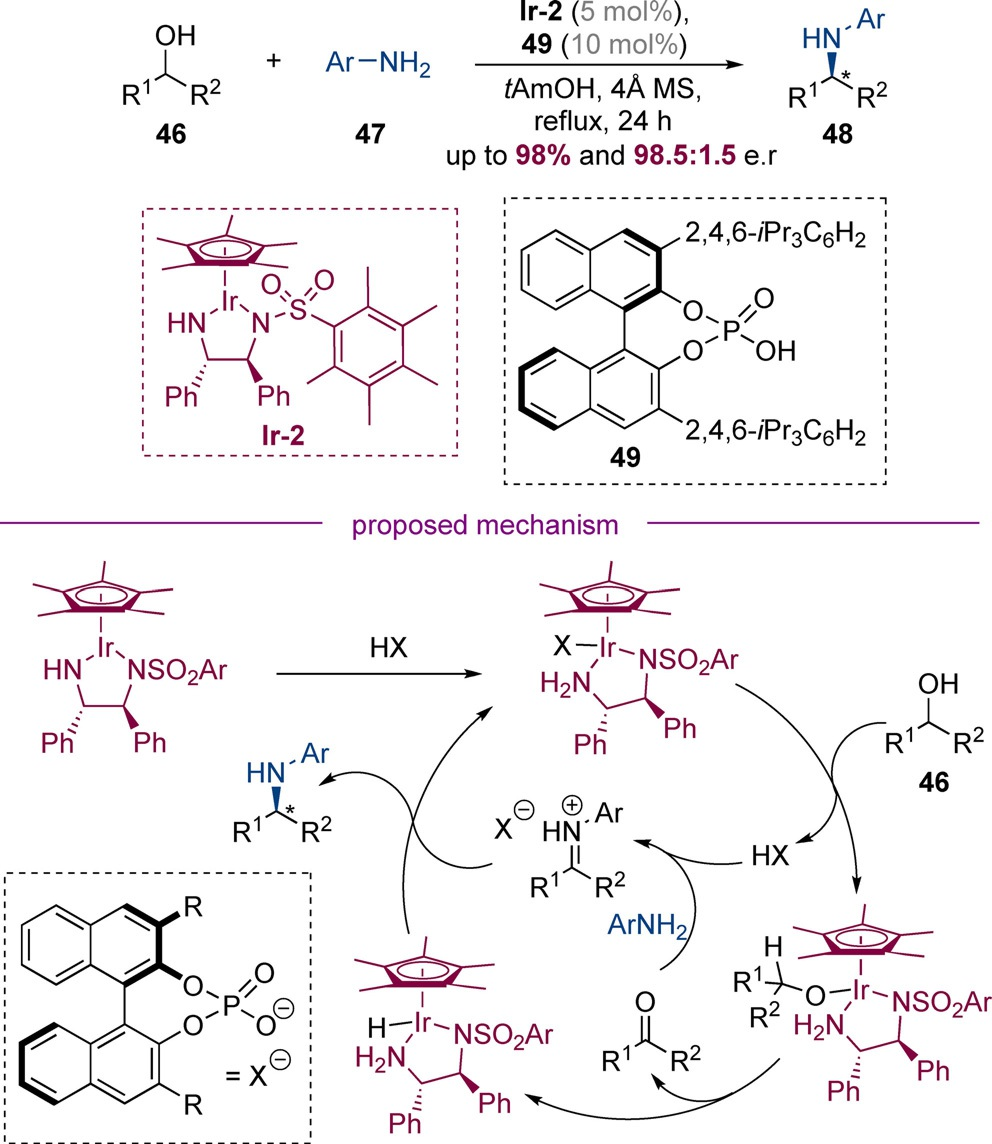
Enantioselective amination of racemic alcohols via cooperative catalysis by a chiral iridium complex and a chiral phosphoric acid.

The concept of employing a chiral transition‐metal catalyst to perform asymmetric hydrogen‐borrowing alkylation of amines has been pioneered by Zhao and co‐workers, who in 2014 reported a dual‐catalytic system that employed a chiral iridium catalyst **Ir‐2** in conjunction with chiral Brønsted acid **49** (Scheme 15).^23^ These conditions enabled the alkylation of anilines **47** with racemic secondary alcohols **46** in high yields and excellent enantioselectivities. The use of a chiral Brønsted acid was essential for both yield and enantioselectivity; both the chirality of the iridium catalyst and phosphoric acid catalyst were important in the process and high enantioselectivities were only observed for the matched case—when the acid was removed entirely, no conversion was observed. Mechanistically, it was proposed that the iridium complex **Ir‐2** is first protonated by the phosphoric acid. The resulting iridium phosphate undergoes ligand exchange with the alcohol followed by oxidation to generate the corresponding ketone, which in the presence of the acid catalyst condenses with the aniline. The resulting protonated iminium species is sufficiently reactive to be reduced by iridium hydride, generating the enantioenriched amine product and regenerating the active catalyst.

**Scheme 16:**
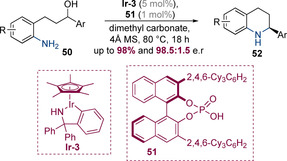
Enantioselective synthesis of tetrahydroquinolines via cooperative catalysis by an achiral iridacycle and a chiral phosphoric acid.

The Zhao group subsequently discovered that chiral phosphoric acids alone can induce high levels of enantioselectivity in intramolecular hydrogen‐borrowing alkylation reactions (Scheme 16).^24^ An achiral Ir catalyst **Ir‐3** in conjunction with chiral phosphoric acid catalyst **51** and could be employed to prepare a range of substituted tetrahydroquinolines **52** in excellent yields with enantioselectivities of up to 98.5:1.5 e.r.

**Scheme 17:**
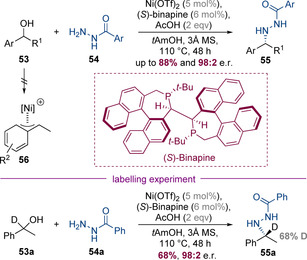
Enantioselective nickel‐catalysed *N*‐alkylation of benzhydrazides.

Tang, Zhou and co‐workers have developed an earth‐abundant catalyst system employing Ni(OTf)_2_ with (*S*)‐binapine to catalyse an asymmetric hydrogen‐borrowing reaction between racemic benzylic alcohols and benzhydrazides (Scheme 17).^25^ The products could be converted to the free benzylamines by reduction with SmI_2_ or Raney nickel. Non‐asymmetric examples employing aliphatic alcohols as substrates were also successful, suggesting that the reaction operates via a “true” hydrogen pathway, as opposed to an alternate mechanism involving a η^3^‐benzylnickel species **56**. Deuterium labelling studies using α‐deuterated benzyl alcohol gave results that corroborated a hydrogen‐borrowing pathway; the resulting benzylamine product only had 68 % deuterium incorporation—this deuterium loss is consistent with in situ washout of deuterium via H/D exchange of a cationic Ni‐D intermediate with protons of acids and alcoholic solvents.

**Scheme 18:**
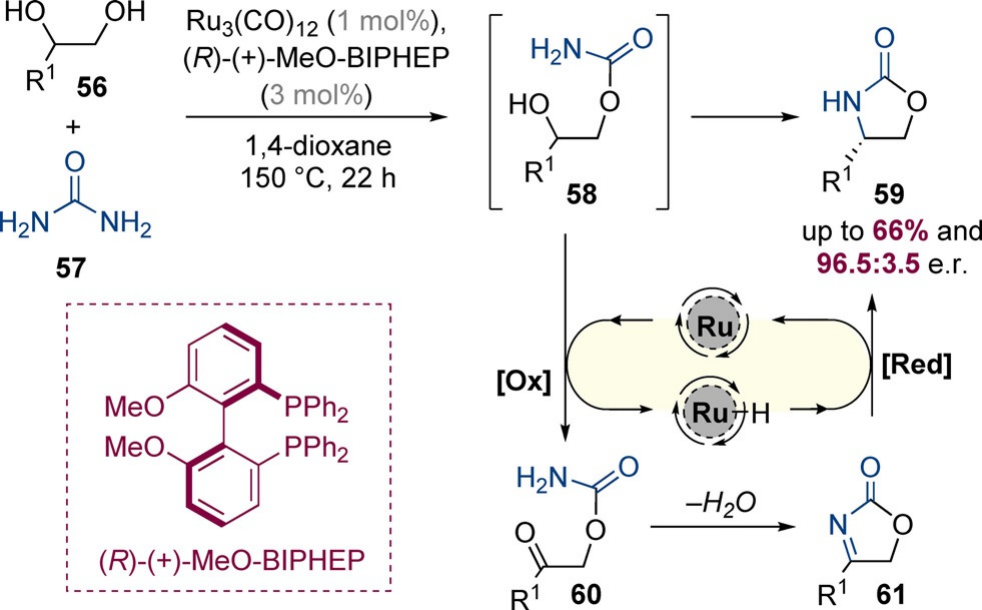
Enantioselective ruthenium‐catalysed synthesis of oxazolidin‐2‐ones from urea and racemic diols.

Beller and co‐workers have developed a highly enantioselective hydrogen‐borrowing reaction that enables the synthesis of chiral oxazolidin‐2‐ones **59** from 1,2‐diols and urea (Scheme 18).^26^ The process is catalysed by a combination of commercially available Ru_3_(CO)_12_ and (*R*)‐(+)‐MeO‐BIPHEP, and is thought to proceed via initial nucleophilic substitution of urea with the less sterically hindered primary hydroxyl group of the diol. This generates amino alcohol **58** in situ, which then undergoes a unimolecular asymmetric hydrogen‐borrowing alkylation. The enantio‐determining step is asymmetric reduction of acyl imine **61**, which proceeds in up to 96.5:3.5 e.r. This high level of enantioselectivity is particularly remarkable given the high reaction temperatures required for this reaction (150 °C).

**Scheme 19:**
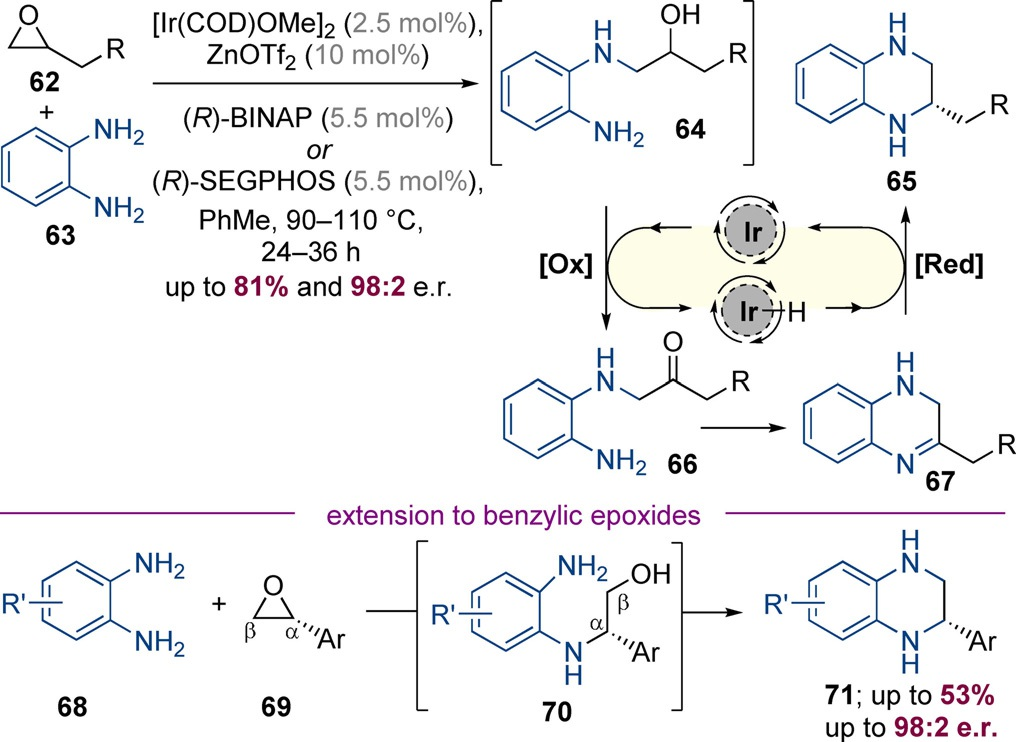
Enantioselective iridium‐catalysed synthesis of tetrahydroquinoxalines through relay epoxide opening/amination.

Very recently, Zhang, Xia, Zhao and co‐workers reported a highly enantioselective Ir^I^‐bisphosphine‐catalysed process in which racemic epoxides and 1,2‐diaminobenzenes were converted to enantioenriched tetrahydroquinoxalines (Scheme 19).^27^ That epoxides instead of more conventional alcohols were used as the non‐nitrogen‐containing partner makes this finding particularly noteworthy. The reaction is initiated by Zn(OTf)_2_‐catalysed epoxide opening, which occurs at primary end of the epoxide to generate a 1,5‐amino alcohol which cyclizes via an enantioselective hydrogen‐borrowing alkylation. Although numerous aliphatic epoxides reacted efficiently, a preliminary experiment involving an aryl‐substituted epoxide gave nearly racemic product. This result was rationalized via an inversion in the regioselectivity of Lewis acid mediated epoxide opening—epoxide opening at the benzylic position would be expected to afford racemic product. However, this problem could be solved by using enantiopure aryl‐substituted epoxides in conjunction with a matched chiral catalyst.

#### Controlling stereocenters adjacent to the site of C−N bond formation

The examples discussed thus far have predominantly focused upon hydrogen‐borrowing reactions which control the stereogenic center which is formed at the site of C−N bond formation (i.e., by stereoselective reduction). However, several additional processes have been developed which enable control over a stereogenic center located adjacent to the amination site.

**Scheme 20:**
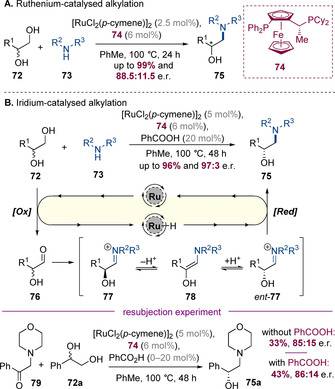
Ruthenium‐catalysed asymmetric amination of racemic 1,2‐diols.

The first such example was reported by Oe and co‐workers, who developed an asymmetric hydrogen‐borrowing approach for the synthesis of β‐amino alcohols from 1,2‐diols (Scheme 20 A).^28^ This reaction was mediated by [RuCl_2_(*p*‐cymene)]_2_ in conjunction with a chiral Josiphos ligand, which resulted in selective amination at the primary site of the diol. Modest, but promising levels of enantiocontrol were observed at the newly forged β‐stereogenic center (up to 88.5:11.5 e.r.). Zhao, Zhang and co‐workers subsequently reported that introduction of an achiral Brønsted acid additive significantly boosts the enantioselectivity of the process (Scheme 20 B).^29^ A wide range of β‐amino alcohols and amines could be reacted in excellent yields and with very high levels of enantioselectivity (up to 97:3 e.r.). Mechanistically, the Zhao group proposed a pathway involving oxidation of the diol followed by condensation of the resulting aldehyde with the amine. The resulting iminium intermediate **77** could rapidly equilibrate with its enantiomer *ent*‐**77** by reversible deprotonation/reprotonation via the intermediacy of enamine **78**. The chiral ruthenium hydride can then selectively reduce *ent*‐**77** (rather than **77**) leading to efficient dynamic kinetic asymmetric amination.^30^ The authors suggest that benzoic acid accelerates the iminium racemization pathway. Interestingly, in their initial publication, Oe and co‐workers proposed a different mechanism involving enantio‐determining reduction of a keto‐amine (e.g. **79**). To rule out this pathway in their acid‐catalysed process, Zhao, Zhang and co‐workers synthesized and resubjected amino ketone **79** to the standard conditions. The product was obtained in significantly lower enantioselectivity (both with and without benzoic acid), implying that this alternative ketone reduction pathway is not operative.

**Scheme 21:**
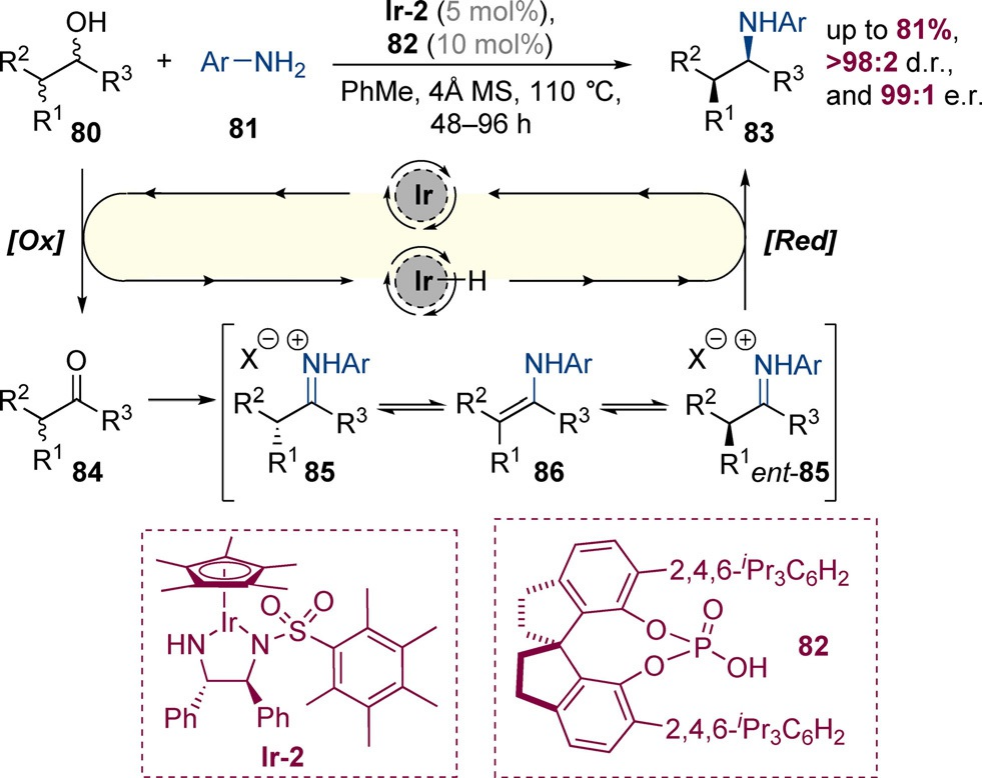
Dynamic kinetic asymmetric amination of alcohols via cooperative catalysis.

The use of asymmetric hydrogen borrowing to control multiple stereocenters is also mechanistically intriguing—such a process would doubly stereoconvergent and could funnel up to four diastereomers towards a single product that is both diastereomerically and enantiomerically pure. Such a concept underpins the Zhao group's work on the asymmetric amination of alcohols to form α,β‐branched amines (Scheme 21).^31^ This reaction is proposed to proceed via a similar pathway to the analogous reaction of 1,2‐diols, but in this case, the chiral iridium catalyst simultaneously controls the β‐stereogenic center by dynamic asymmetric transformation along with the facial selectivity of iminium reduction. Very high levels of both diastereo‐ and enantioselectivity were observed in this process. The substrate scope was remarkably broad and a wide variety of aromatic, aliphatic and alkoxy substituents could successfully be introduced at the β‐position (R^1^/R^2^).

**Scheme 22:**
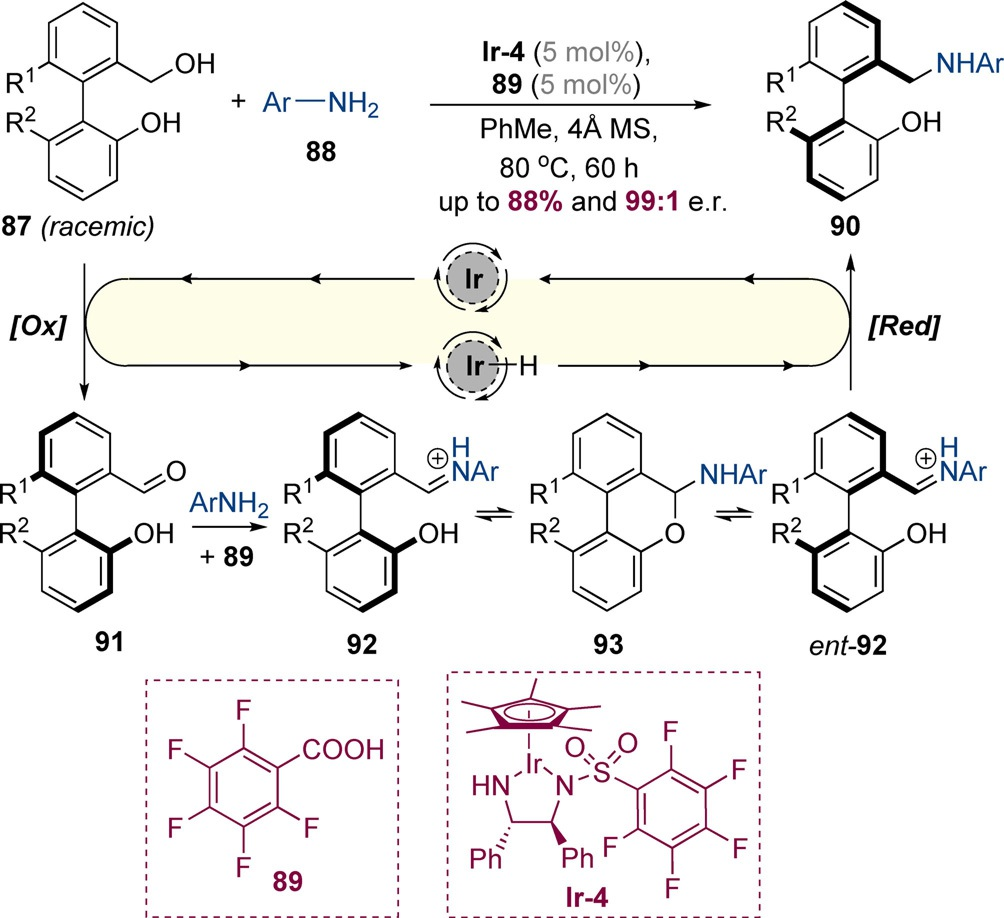
Iridium‐ and acid‐catalysed atropselective amination.

It is also possible to employ asymmetric hydrogen‐borrowing catalysis to control an adjacent axis of chirality. For example, Zhang and Wang have reported a highly atropselective amination of biaryl alcohols **87** (Scheme 22).^32^ This reaction is mediated by a chiral iridium(III) catalyst in conjunction with an achiral Brønsted acid. The reaction is proposed to proceed via iridium‐mediated oxidation of the racemic biaryl alcohol to the corresponding aldehyde **91**, which then condenses with the amine to form imine **92**. This intermediate can undergo reversible cyclization to from cyclic hemiaminal **93**, which is expected to have a low barrier to biaryl rotation due to the “bridged biaryl” effect discovered by Bringmann.^33^ This allows interconversion between the enantiomers of the biaryl iminium (**92**↔ent‐**92**) enabling a dynamic kinetic resolution in which only one enantiomer undergoes reduction to form **90** with very high enantioselectivity. It was shown that the biaryl amine products have a very high barrier to racemization (Δ*G* ^≠^_rot_=129.0 kJ mol^−1^, *t*_1/2_^rac^=157.6 h at 80 °C).

### Use of enzymes

**Scheme 23:**
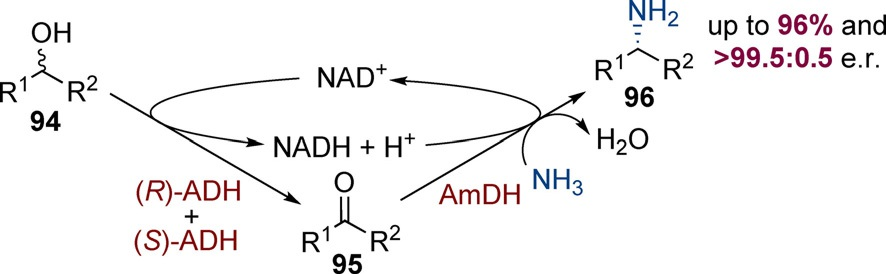
Asymmetric amination of racemic alcohols using enzymes.

Biocatalysis can offer a useful alternative approach for hydrogen‐borrowing amination in which each elementary step is catalysed by an individual enzyme. Such reactions are amenable to low operating temperatures and, in several cases, enzymatic catalysis has been conducted with racemic secondary alcohols to give enantiopure amines.^34^ As a representative example, Turner and co‐workers have reported an elegant enzymatic process for the asymmetric alkylation of ammonia (Scheme 23).^35^ The first step involves a pair of alcohol dehydrogenase enzymes (AD), which oxidise the alcohol to the corresponding ketone along with concomitant conversion of NAD^+^ to NADH and H^+^ (two AD enzymes are required, one to oxidise each enantiomer of the racemic alcohol). The resulting ketone then condenses with ammonia to form an imine, which is reduced by an amine dehydrogenase (AmDH) to generate the chiral amine product. This step also converts NADH back to NAD^+^, thereby completing the catalytic cycle. This method can be employed to prepare an assortment of amines with excellent levels of enantioselectivity. The Turner group and others have subsequently reported exciting developments in this field, including streamlining the process to use a single non‐stereoselective AD enzyme,^36^ and expanding the scope to other amine nucleophiles.^37^ Recently, it has even been shown that amination is possible in *E. coli* cells.^38^ As this chemistry does not involve transition‐metal catalysis, a comprehensive discussion lies outside the scope of this Minireview, but enzymatic chemistry can offer a useful alternative to transition‐metal‐catalysed methods.

## C−C Bond Formation

**Scheme 24:**
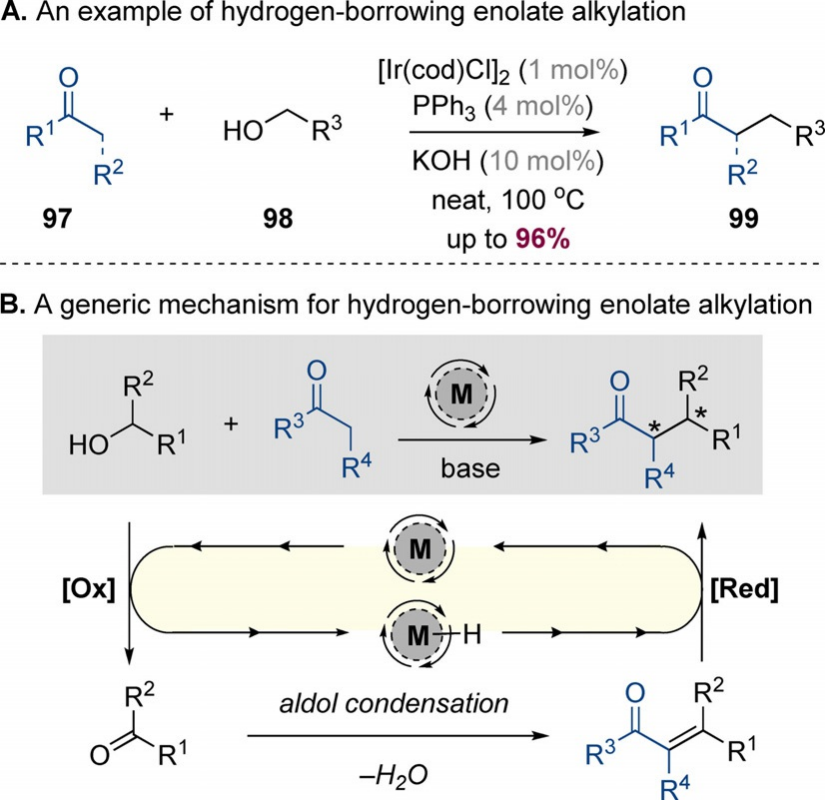
A representative example of enolate alkylation using hydrogen‐borrowing catalysis and a generic mechanism for C−C bond formation.

The formation of carbon‐carbon bonds under hydrogen‐borrowing conditions is a well‐established method that has been successfully applied to the alkylation of ketones, esters and nitriles. As a representative example of a non‐stereoselective process, Ishii and co‐workers have shown that various ketones can undergo α‐alkylation with primary alcohols to form alkylated products **99** in excellent yields (Scheme 24 A).^39^ Mechanistically, these reactions are analogous to the corresponding amine alkylation reactions, proceeding via catalyst‐promoted oxidation of the alcohol to the corresponding carbonyl compound along with formation of a transition‐metal hydride (Scheme 24 B). The aldehyde or ketone then undergoes a base‐mediated aldol condensation with an enolate, followed by conjugate reduction of the resulting enone to deliver the saturated product and regenerate the active catalyst. This section will discuss several strategies for induction of asymmetry including carbonyl reduction, enzymatic catalysis, organocatalysis and catalytic asymmetric enone reduction. These strategies are detailed sequentially in the following sections.

### Asymmetric carbonyl chemistry

**Scheme 25:**
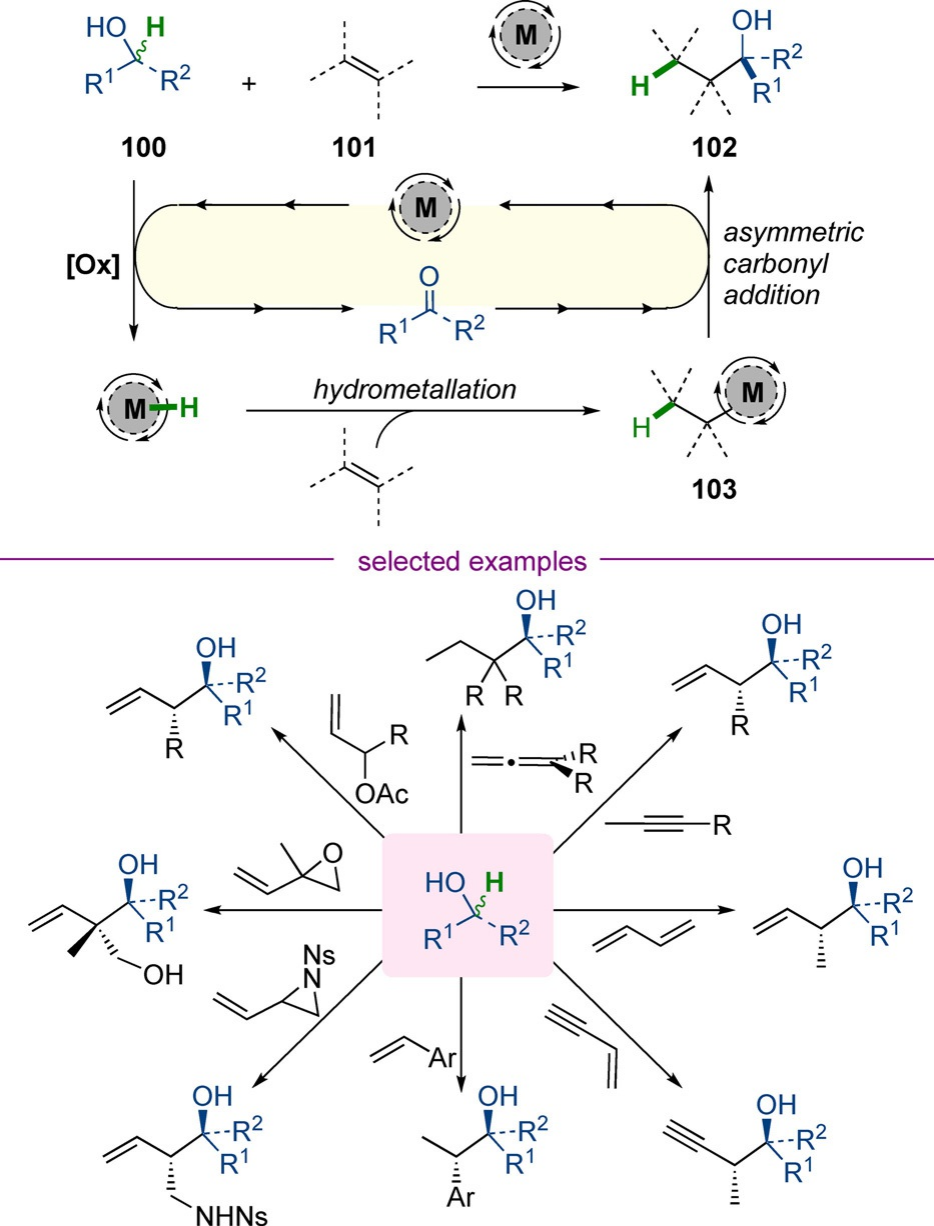
General mechanism and selected examples of redox‐neutral carbonyl addition chemistry developed by Krische and co‐workers.

Krische and co‐workers have pioneered a powerful new approach for asymmetric carbonyl addition which relies upon hydrogen‐borrowing catalysis (Scheme 25). In this chemistry, an alcohol **100** and a π‐unsaturated partner **101** can undergo redox‐neutral coupling, enabling the highly enantioselective synthesis of homologated alcohol products **102**. Mechanistically, this chemistry is distinct from all other examples presented thus far—the process is still initiated by transition‐metal‐mediated oxidation of an alcohol, but the metal hydride which is produced directly reacts with the π‐unsaturated partner, converting it to an organometallic nucleophile **103** in situ. The enantio‐determining step involves recombination of the carbonyl and organometallic partners. The process is remarkably general and has been applied to couplings of alcohols with an extensive array of π‐unsaturated partners (selected examples are shown in Scheme 25 and include allenes, alkynes, dienes, styrenes, and a various allylic electrophiles).^40^ This is a rapidly developing area of research and has previously been reviewed elsewhere.^41^ A full discussion lies outside the remit of this Minireview, but this chemistry represents an extremely useful approach for enantioselective redox‐neutral synthesis.

**Scheme 26:**
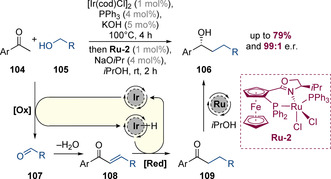
Stepwise process involving asymmetric carbonyl reduction.

Several groups have investigated asymmetric carbonyl reduction within the context of hydrogen‐borrowing catalysis. This area was pioneered by Nishibayashi and co‐workers, who in 2006, reported a one‐pot process for the alkylation of acetophenones (**104**) with primary alcohols (**105**) yielding secondary alcohols (**106**) in good yields and with excellent enantioselectivities (Scheme 26).^42^ The first step of the process is a typical iridium‐catalysed hydrogen‐borrowing alkylation to generate achiral ketone **109**. In the second step, ruthenium‐catalysed asymmetric transfer hydrogenation of ketone **109** with isopropanol as a sacrificial hydride source sets the benzylic stereocenter generating chiral alcohols **106** in up to 99:1 e.r.

**Scheme 27:**
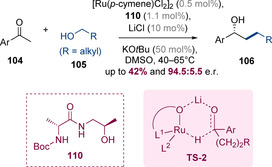
One‐pot tandem hydrogen‐borrowing alkylation‐transfer hydrogenation procedure employing a single ruthenium catalyst.

This method was subsequently developed by Adolfsson and co‐workers.^43^ The authors reported that a single ruthenium catalyst can promote both the hydrogen‐borrowing alkylation and asymmetric reduction step (Scheme 27). A combination of [Ru(*p*‐cymene)Cl_2_]_2_ with chiral ligand **110** gave reduced products **106** at moderate reaction temperatures and with good levels of enantioselectivity. In this case, an excess of the alcohol partner **105** (3 equiv) served as the terminal reductant for the transfer‐hydrogenation step. The authors observed that addition of substoichiometric amounts of lithium chloride increased the reaction rate as well as the enantioselectivity. The positive effect of lithium chloride on the catalytic activity of similar ruthenium‐based transfer hydrogenation catalysts had been established previously and can be rationalized by reaction via transition state **TS‐2** wherein the lithium ion aids the hydride transfer by coordination to both the substrate and catalyst.^44^

**Scheme 28:**
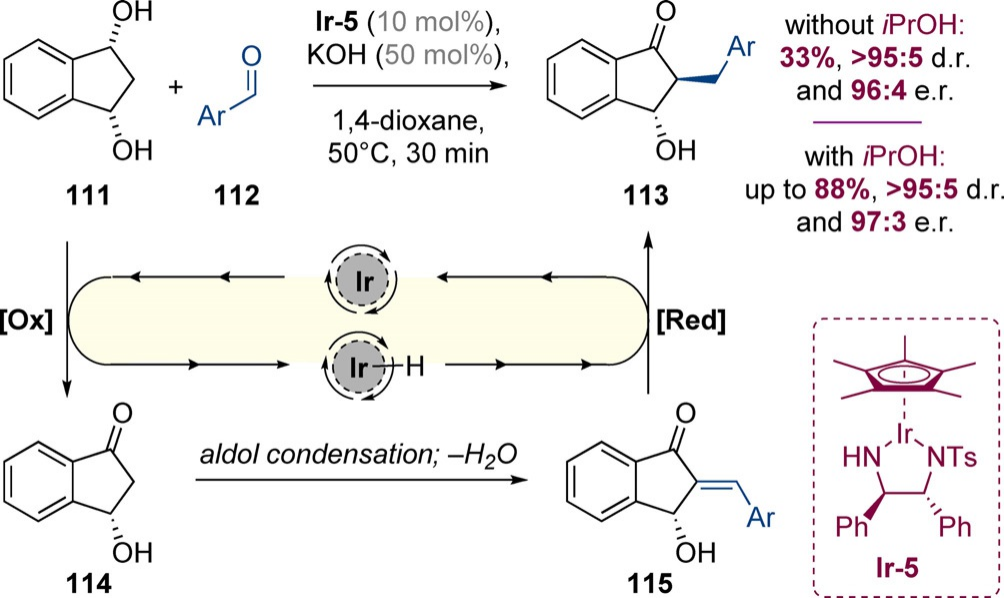
An asymmetric hydrogen‐borrowing alkylation involving desymmetrization of a *meso*‐diol.

Suzuki and co‐workers have reported an interesting hydrogen‐borrowing alkylation between *meso*‐diol **111** and benzaldehydes (Scheme 28).^45^ The authors proposed that the absolute stereochemistry is set in the first step, which involves oxidative desymmetrization of diol **111**. This is followed by a typical hydrogen‐borrowing pathway involving condensation with benzaldehyde and diastereoselective enone reduction. The product **113** was obtained in excellent diastereo‐ and enantioselectivity, but the yield was low (33 %) due to poor conversion in the final reduction step. However, it was discovered that addition of isopropanol as a sacrificial reductant after 30 minutes could ensure that complete reduction takes place enabling the isolation of products **113** in up to 88 % yield, still with very high levels of stereoselectivity.

### Biocatalysis

**Scheme 29:**
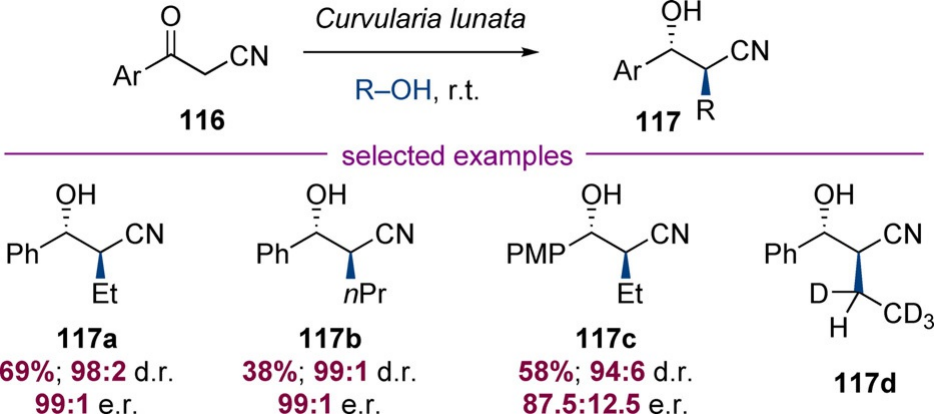
Biocatalysed asymmetric alkylation of cyanoketones.

Several elegant biocatalytic enolate alkylation procedures have been developed. A detailed discussion of this chemistry is beyond the remit of this Minireview, but this can be a useful alternative to transition‐metal based approaches. As a representative example, Gotor and co‐workers have reported a hydrogen‐borrowing alkylation of α‐cyano ketones (**116**) with primary alcohols catalysed by the fungus *Curvularia lunata* (Scheme 29).^46^ The products were obtained in good to excellent stereoselectivity and useful yields from a preparative synthetic viewpoint. The observation that tetradeuterated product **117 d** was isolated when hexadeuteroethanol was employed supports the proposed hydrogen‐borrowing mechanism for this transformation.

### Conjugate addition

**Scheme 30:**
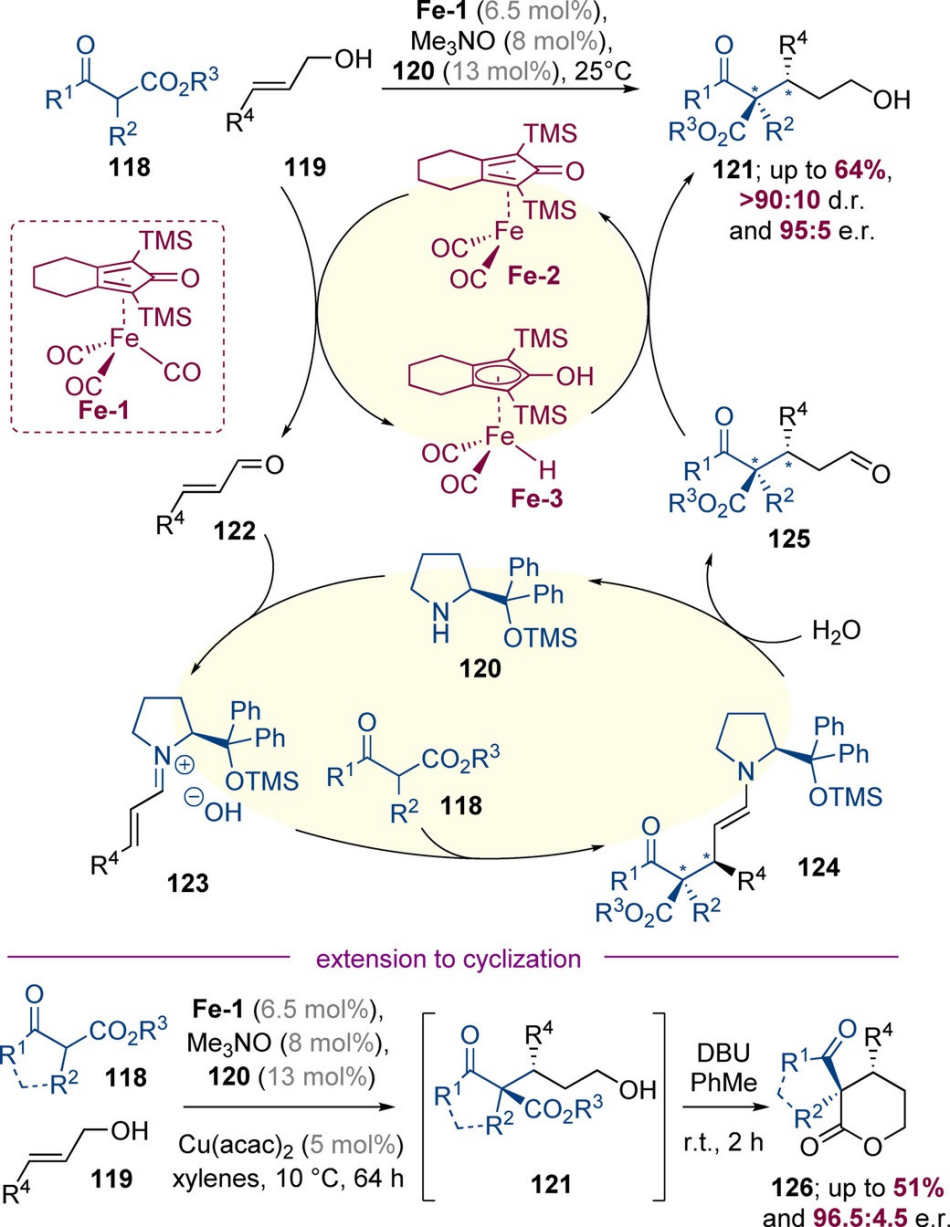
Dual catalytic reaction of allylic alcohols with β‐ketoesters.

Quintard, Rodriguez, and co‐workers have pioneered a fundamentally different approach to induce enantioselectivity in hydrogen‐borrowing catalysed reactions. The authors reported a dual‐catalytic process mediated by a combination of an achiral iron complex along with a chiral proline‐derived organocatalyst (Scheme 30).^47^ Under these conditions, β‐ketoesters **118** could be reacted with primary allylic alcohols (**119**) to efficiently generate chiral alcohols **121** with high levels of diastereo‐ and enantioselectivity. This reaction is thought to operate by initial activation of Knölker complex **Fe‐1** with trimethylamine *N*‐oxide to give catalytically active species **Fe‐2** along with CO_2_ and trimethylamine as by‐products. This iron complex can oxidise the allylic alcohol **119** to form α,β‐unsaturated aldehyde **122** along with iron hydride **Fe‐3**. In a second catalytic cycle the aminocatalyst **120** condenses with aldehyde **122** to form iminium **123**, followed by asymmetric conjugate addition of the β‐ketoester nucleophile. Hydrolysis of addition product **124** results in aldehyde **125** and regenerates organocatalyst **120**. Finally, facile reduction of the saturated aldehyde by **Fe‐3** closes the hydrogen‐borrowing cycle and delivers the chiral alcohol product **121**. It was subsequently shown that the enantioselectivity of this reaction can be enhanced by the addition of Cu(acac)_2_ as an additional co‐catalyst.^48^ Treatment of the hydroxyester products (**121**) with DBU (1,8‐diazabicycloundec‐7‐ene) resulted in lactonization to generate a range of enantioenriched cyclic derivatives (**126**).

**Scheme 31:**
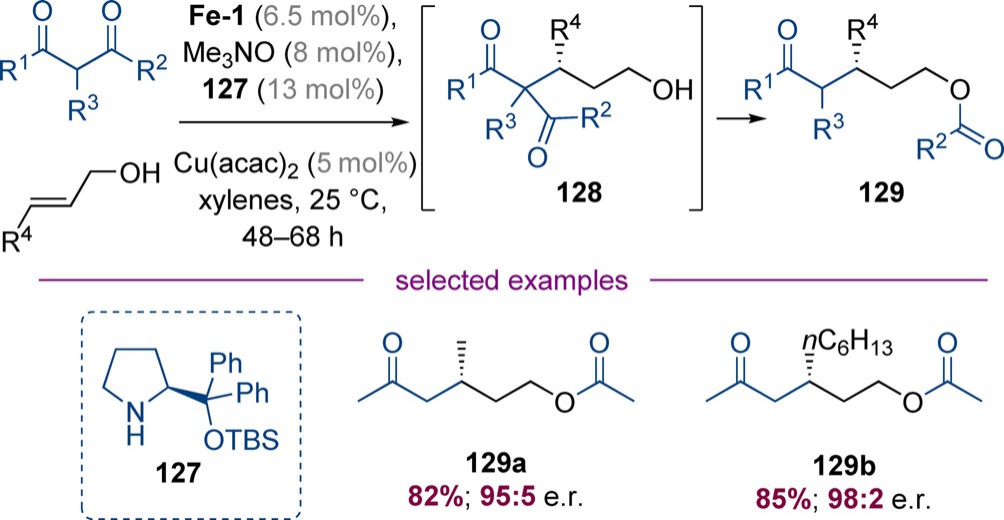
Asymmetric functionalization of 1,3‐diketones with allylic alcohols.

Quintard, Rodriguez, and co‐workers have also reported that 1,3‐diketones can be employed as nucleophiles under similar reaction conditions (Scheme 31).^49^ In this case, the intermediate hydroxyketone intermediates **128** underwent a spontaneous C‐ to O‐acyl shift to deliver synthetically useful protected alcohols **129**. A series of examples were prepared in high yields with excellent levels of enantioselectivity (for example **129 a**–**129 b**). The authors demonstrated that the reduction of saturated aldehydes, by iron hydride **Fe‐3** is much more facile than the corresponding reaction with unsaturated aldehydes, suggesting a synergistic link between the two catalytic cycles.

**Scheme 32:**
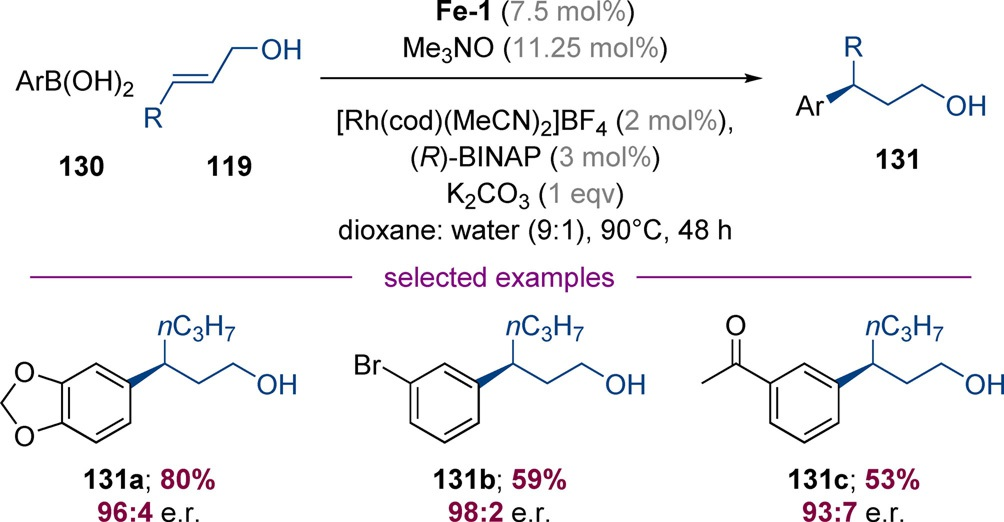
Bi‐catalytic functionalization of allylic alcohols with boronic acids.

Very recently, Dydio and co‐workers reported a related method in which primary allylic alcohols were again transiently activated via oxidation with Knölker's complex (Scheme 32).^50^ In this case, the α,β‐unsaturated aldehyde intermediates were captured in a highly enantioselective Rh‐BINAP‐catalysed conjugate addition reaction with aryl boronic acids, enabling the synthesis of γ‐functionalized alcohols in good yields and excellent enantioselectivities. It was also shown that RuH_2_(PPh_3_)_4_ can be used in place of Knölker's complex and in some cases gives higher yields and selectivities, whereas **Fe‐1** tolerates a wider substrate scope.

### Selective enone reduction

The most direct method to control absolute stereoselectivity within hydrogen‐borrowing enolate alkylation reactions would be to control the facial selectivity of the final enone reduction step. However, remarkably few examples of such asymmetric processes have been reported. There are two problems which have limited the development of such asymmetric reactions: (i) the strongly basic conditions required to promote aldol condensation typically lead to racemization at the α‐stereogenic center; (ii) it is essential to control the geometry of the enone intermediate in order to obtain high levels of enantioselectivity. However, a handful of asymmetric enolate alkylation processes have been developed that overcome these challenges.

**Scheme 33:**
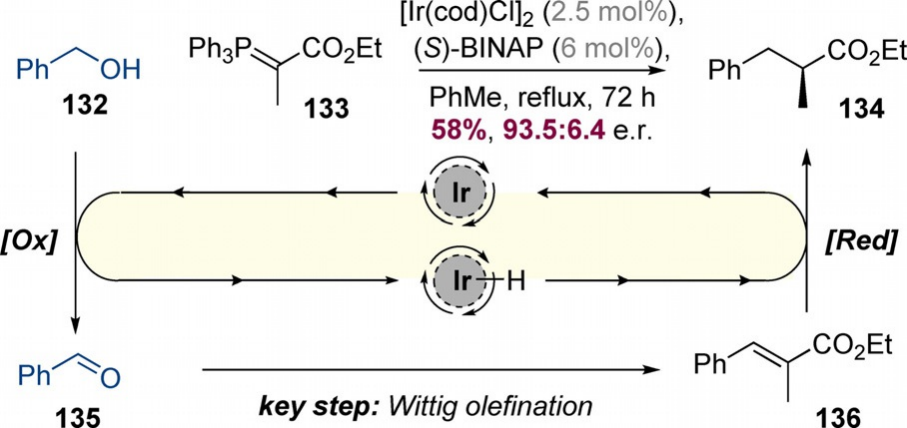
A pioneering example involving a Wittig reaction embedded within an asymmetric hydrogen‐borrowing process.

This area was pioneered by Williams and co‐workers, who in 2007 reported an asymmetric hydrogen‐borrowing reaction between benzyl alcohol and stabilized Wittig ylide **133** (Scheme 33).^51^ This reaction operates via a similar mechanism to a standard hydrogen‐borrowing process, except that the aldol step is replaced by a Wittig olefination. This was key to the success of the process as it ensured that enone intermediate **136** was formed as a single *E* isomer and also enabled the reaction to proceed in the absence of base, limiting the possibility of racemization of the newly formed α‐stereogenic center in **134**. Employing an Ir^I^‐BINAP system, **134** was isolated in 58 % yield with very high enantioselectivity (93.5:6.5 e.r.).

**Scheme 34:**
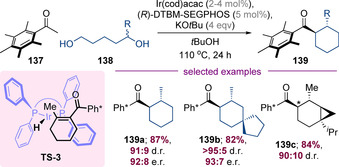
[5+1] strategy for the synthesis of substituted acyl‐cyclohexanes.

Donohoe and co‐workers have shown that pentamethylphenyl (Ph*) ketones can be alkylated with a wide variety of primary or secondary alcohols and diols.^52^, ^53^ The Ph* group is pivotal for these transformations—because the aryl group is orthogonal to the carbonyl, the *ortho*‐methyl groups protect it from undesired reduction and homodimerization processes. Moreover, the Ph* group can readily be converted to a wide range of functional groups (e.g., esters, amines, acids and alcohols) by retro‐Friedel–Crafts acylation.^52^ Donohoe and co‐workers recently reported that pentamethylacetophenone **137** can undergo a highly enantioselective hydrogen‐borrowing annulation with diols **138** to synthesize enantioenriched substituted cyclohexanes **139** (Scheme 34).^54^ It is thought that the enantiodetermining step involves iridium hydride mediated reduction of a cyclic enone intermediate via transition state **TS‐3**. The cyclic nature of the enone intermediate enforces a single alkene geometry, thereby enabling highly enantioselective reduction to form acyl‐cyclohexane products such as **139 a**–**139 b**. The Donohoe group had previously shown that enantiopure γ‐substituted 1,5‐diols react without racemization,52c, 52d and it was found that by matching or mismatching the chiral ligand complex diastereomerically enriched products such as **139 c** could be synthesized in very high yield and diastereoselectivity.

**Scheme 35:**
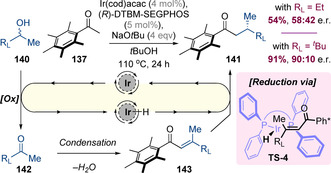
Asymmetric synthesis of β‐substituted acyclic Ph* ketones.

Donohoe and co‐workers subsequently reported that pentamethylacetophenone **137** can also undergo asymmetric hydrogen‐borrowing alkylation with methyl‐substituted secondary alcohols **140** (Scheme 35).^55^ This process operates via a typical hydrogen‐borrowing mechanism and the key step therefore involves asymmetric reduction of acyclic enones **143**. For high enantioselectivity to be observed, it was essential for the second alcohol substituent (R_L_) to be significantly larger than a methyl group—for example, enantioselectivities of 58:42 e.r. and 90:10 e.r. were obtained for R_L_=Et and *t*Bu, respectively. It was proposed that when the substituents are sterically well‐differentiated, a single geometrical isomer of the enone intermediate **143** could form, which was essential to achieve efficient asymmetric induction. The absolute stereochemical outcome of the reduction step was analogous to the related reduction of cyclic enones discussed above (i.e. via **TS‐4**).

## Conclusion

Transition‐metal‐catalysed asymmetric hydrogen‐borrowing catalysis is rapidly emerging as a powerful method for the formation of both C−N and C−C bonds. Various strategies have been developed to achieve such reactions, including asymmetric reduction, dynamic kinetic resolution, enantiospecific reactions and desymmetrization. Metals from across the *d*‐block can be employed in these reactions, and examples are presented involving catalysis by iridium, ruthenium, rhodium, iron, nickel and manganese complexes, including several examples which also employ organic co‐catalysts. These methods enable efficient access to a wide range of useful enantiopure amine and carbonyl containing materials. The field of asymmetric hydrogen‐borrowing catalysis is currently in a phase of rapid development and the possibility for further advances and applications of these processes is very exciting.

## Conflict of interest

The authors declare no conflict of interest.

## Biographical Information

*Timothy Kwok obtained his BSc (Hons) in Chemistry and graduated as Valedictorian of his seniors‘ cohort from the National University of Singapore in 2016. For his BSc final year project, he worked under Assoc. Prof. Yulin Lam on the synthesis of fluorogenic probes to monitor the activity of the enzyme cytosolic phospholipase A2. In 2016, he received a Clarendon Fund Scholarship to join the T. J. Donohoe group, where he works on the total synthesis of pectenotoxin‐4 as his DPhil project*.

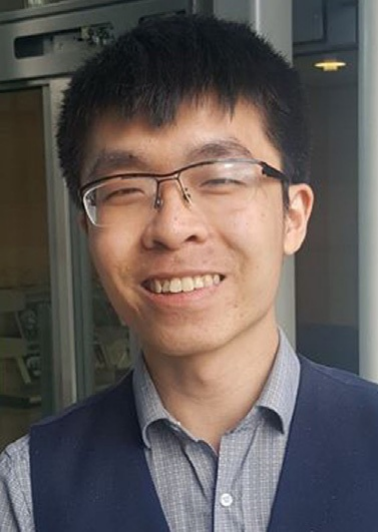

## Biographical Information

*Oskar Hoff received his B.Sc. (2013) and M.Sc. (2015) from Graz University of Technology working for Assoc. Prof. Tanja Wrodnigg. During his degree he visited the University of Syracuse, USA and the Ferrier Research Institute in Wellington, New Zealand. In 2015 Oskar did an industrial placement at Hoffmann‐La Roche in Basel, Switzerland. In 2016 he joined the Synthesis for Biology and Medicine Centre for Doctoral Training (SBM CDT) in Oxford, where his research is focused on the total synthesis of (+)‐Lophotoxin with Prof. Timothy J. Donohoe*.

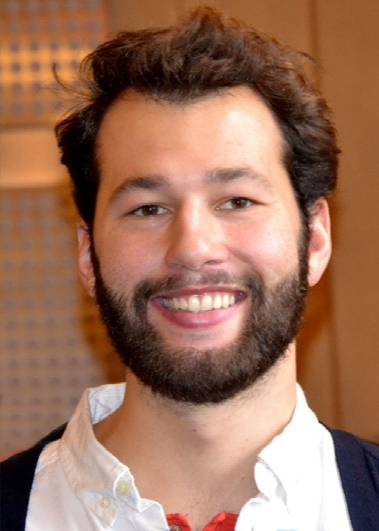

## Biographical Information

*Roly J. Armstrong graduated with an MSci in Natural Sciences from Pembroke College, Cambridge (2011) spending his final year working in the laboratory of Professor Steven Ley FRS. He subsequently moved to Merton College, Oxford to carry out a DPhil under the supervision of Professor Martin Smith (2011–2015). In 2015, he joined the group of Professor Varinder Aggarwal FRS at the University of Bristol as a postdoctoral research associate. He is currently a Junior Research Fellow at University College, Oxford where his research is focussed on stereoselective hydrogen borrowing catalysis*.

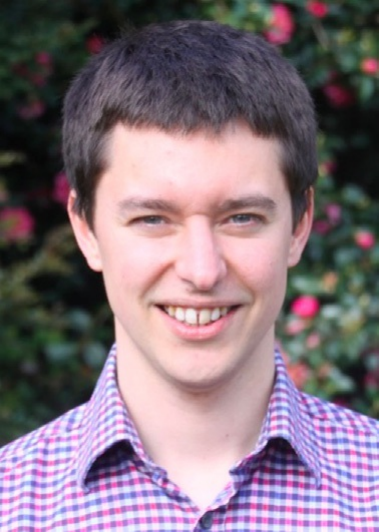

## Biographical Information

*Timothy J. Donohoe obtained his D.Phil. with Professor S. G. Davies at the University of Oxford (1992). Following a postdoctoral stay with Professor P. D. Magnus FRS in the USA, he joined the University of Manchester in 1994 as a lecturer and was promoted to Reader in 2000. In 2001, he joined the Dyson Perrins Laboratory, Oxford, as a Lecturer in Chemistry and a Fellow of Magdalen College. In 2004, he was appointed Professor of Chemistry at Oxford University. His research interests encompass catalysis, asymmetric synthesis, total synthesis, and redox reactions*.